# Implementing a continuum of care model for older people—results from a Swedish case study

**DOI:** 10.5334/ijic.665

**Published:** 2011-11-18

**Authors:** Anna Dunér, Staffan Blomberg, Henna Hasson

**Affiliations:** Department of Social Work, University of Gothenburg, P.O. Box 720, SE-405 30 Göteborg, Sweden; Vårdal Institute, The Swedish Institute for Health Sciences, P.O. Box 187, SE- 221 00 Lund, Sweden; Vårdal Institute, The Swedish Institute for Health Sciences, P.O. Box 187, SE- 221 00 Lund, Sweden; School of Social Work, Lund University, P.O. Box 23, SE-221 00 Lund, Sweden; Lund University School of Economics and Management, Department of Business Administration, Lund, Sweden Vårdal Institute, The Swedish Institute for Health Sciences, P.O. Box 187, SE- 221 00 Lund, Sweden; Karolinska Institute, Department of Learning, Informatics, Management and Ethics, Medical Management Centre (MMC), Stockholm, Sweden

**Keywords:** older people, continuum of care, integrated care, implementation, qualitative methods, Sweden

## Abstract

**Introduction:**

There is a need for integrated care and smooth collaboration between care-providing organisations and professions to create a continuum of care for frail older people. However, collaboration between organisations and professions is often problematic. The aim of this study was to examine the process of implementing a new continuum of care model in a complex organisational context, and illuminate some of the challenges involved. The introduced model strived to connect three organisations responsible for delivering health and social care to older people: the regional hospital, primary health care and municipal eldercare.

**Methods:**

The actions of the actors involved in the process of implementing the model were understood to be shaped by the actors' understanding, commitment and ability. This article is based on 44 qualitative interviews performed on four occasions with 26 key actors at three organisational levels within these three organisations.

**Results and conclusions:**

The results point to the importance of paying regard to the different cultures of the organisations when implementing a new model. The role of upper management emerged as very important. Furthermore, to be accepted, the model has to be experienced as effectively dealing with real problems in the everyday practice of the actors in the organisations, from the bottom to the top.

## Introduction

In this study, the process of implementing a continuum of care model in the context of local Swedish eldercare is described and analysed. In Sweden, as in other countries, the needs of the ageing population impose challenges on eldercare [1–3]. The need for integrated care and smooth collaboration between various care-providing organisations and professions to create a continuum of care for older people has been emphasised in Sweden as well as internationally [2, 4–8]. However, collaboration between organisations and professions is often problematic due to different organisational as well as professional logics and cultures [9–12], conflicting legislations and professional knowledge and value bases, along with conflicting economic and other interests of the organisations and professions involved [8, 12, 13]. The situation calls for further studies promoting an evidence-based practice [5, 14, 15].

Sweden is regarded as a universal welfare state in terms of its systems of benefits and services linked to the lifetime needs of its citizens. The formal goals of the Swedish welfare system comprise universalism and extensive coverage [16]. Since 1992, the municipalities have the overall responsibility for social care and services for older people and people with disabilities, including special housing [17]. However, the division of responsibility for health care is complex and varies depending on local agreements [18]. Health care provided by both county councils/regions and municipalities. The county councils are responsible for all health care except for older people or people with disabilities living in special housing. Within the county councils the regional/local hospitals are responsible for acute medical care that requires hospital admission, and primary health care centres are responsible for outpatient care. Municipal healthcare is in charge of health care for those living in special housing, and after local agreements for home nursing for those living in their own homes. This calls for integration and collaboration between all involved care providers. In Sweden, ‘Health care chains’, understood as “coordinated activities in the health care systems linked together to achieve a final result of good quality for the patient”, have become an important part of integrated care [19, 20, 21]. Different initiatives have been performed at the local level, however, national evaluations point to deficits in the provision of integrated care [4–6].

When introducing new initiatives to accomplish integrated care, Glendinning [8] emphasises the importance of distinguishing between short-term implementation problems and their solutions, and fundamental flaws that may impose barriers to the performance of integrated care for older people. She concludes that organisational integration therefore needs to be supplemented with extensive engagement in supporting change among those involved in performing integrated care, in order to relinquish traditional professional and organisational roles and status conditions. Åhgren and Axelsson [22] report that there are three major determinants of successful performance of integrated health care chains. These are professional dedication, legitimacy and confidence, and space to develop trust and motivation among the involved actors. Both Glendinning, Åhgren and Axelsson recommend a move towards bottom-up approaches to integration [8, 22]. Still, there is a need for further studies concerning the process of implementing new initiatives aimed at providing a continuum of care for older people.

### Aim and research questions

This study aims to examine the process of implementing a new continuum of care model in a complex organisational context, and illuminate some of the challenges involved. The following research questions are posed:

- How do the actors involved at different organisational levels understand the model?
- What commitment do they express to the model?
- How do they perceive their ability to put the model into practice?

### Analytical framework

In the process of implementing a new policy or model in health and social care, the senders may be either policy makers at different levels or the scientific community. The receivers may be practitioners in human services, civil servants and other practitioners [23]. Implementation may be regarded as a straightforward, planned process and any problems that occur are caused by ‘improper’ implementation. Therefore, the receivers' fidelity to the model is crucial for its ‘proper’ implementation [23, 24]. However, implementation of new policies or models may also be regarded as a response to the technical and institutionalised environment of the organisations [25], and their transformation at the operative level depends on the local context [21, 23]. Professional discretion and the distribution of power between the actors involved, as well as the organisations' adaptation to the environment [25, 26–28], shape the implementation process.

In this article, we took a bottom-up approach to implementation emphasising the importance of discretion for the actions of the staff responsible for delivering social and health care according to the new model. According to Lipsky's theory of street-level bureaucracy, discretion is fundamental in professional practice in human service organisations. Discretion is based on three conditions; the staff must interpret rules that are often conflicting and vague, the circumstances and needs of the people receiving services may be both unique and unpredictable, and the staff is presumed to perform their task based on professional expertise providing latitude to personal discretion [26].

In the study of the linkage between the implemented model and the actual performance, we have used a model enabling us to analyse the actions of the actors involved. The process of implementing the model are understood as being shaped by the actors' understanding (‘I understand’), commitment (‘I will’) and ability (‘I can’) [27, 29]. ‘Understanding’ concerns the actors' perceptions of the policy or the model that is being implemented, and whether, and how well, they are informed about the existence and content of what is being implemented. ‘Commitment’ relates to the actors' more or less conscious and explicit preferences and their willingness to support the introduced intervention. By ‘ability’ is meant the actors' capability to work according to the new model. The concept takes into account both the actor's ability to make decisions and shape her/his environment accordingly and the resources needed to put the model into practice [27].

## Methods

The study was conducted in a city in south-western Sweden, with approximately 60,000 inhabitants, where a continuum of care model was introduced and tested. The model strived to connect three organisations responsible for delivering health and social care to older people: the regional hospital, primary health care and municipal eldercare. Although primary care was invited to participate in the study on several occasions, no overall involvement was obtained.

This article is based on 44 qualitative interviews performed on four occasions between 2008 and 2010 with 26 key actors at three organisational levels within the organisations involved (Table 1). The respondents were interviewed on one to four occasions each. At the upper management level, the interviewees were the heads of geriatric, medical and emergency care departments at the regional hospital, the head of primary health care, the heads of home care, the social care office and the health care and rehabilitation department at the municipality. The interviewees at the management level were the department director at the emergency ward of the hospital, the managers for health care, and rehabilitation and social workers. The respondents at the operative level were the geriatric nurses at the emergency ward of the hospital and nurses from other hospital wards involved in the project, the CMs, and other members of the municipal team, e.g., social workers, and occupational and physiotherapists.

The interviews each lasted 30 minutes to 1 hour. Interviews were audio-recorded and transcribed verbatim. The analysis proceeded from open to focused coding in several steps [30]. Discussions among the authors took place in each step of the analysis. In the first step, we took an empirically oriented approach using the concepts understanding, commitment and ability as a general sense of reference, or as sensitising concepts [31]. Meaning units relevant to the aim of the study were selected from the interviews and condensed. In the second step, we identified and organised themes according to the sensitising concepts and the identified phases of the implementation process (Tables 2, 3, and 4). In the conclusive discussion we related the findings to our analytical framework and existing knowledge. We emphasise that neither the sensitising concepts nor the empirical themes should be regarded as mutually exclusive; they may overlap. The analysis was interwoven with other steps of the research process, as characteristic of most qualitative research [32].

This research forms part of the randomised control trial (RCT) study ‘Continuum of Care for Frail Elderly People’ evaluating the effect and implementation of a continuum of care model targeting frail older people [3, 33]. It is performed by an interdisciplinary research group at the Vårdal Institute, a national environment for research and development in the field of health care and social service. The research group consists of both researchers studying the effects of the intervention and researchers studying the implementation process (the authors of this paper). The results will be used to formulate a local programme for a continuum of care for this population group. The study was approved by the Regional Ethics Board in Gothenburg, Sweden (study code 413-08).

The introduced continuum of care model was designed in cooperation between representatives from the care-providing organisations and intervention-researchers from the Vårdal Institute. The development of the model is based on experiences from practitioners and managers in the care providing organisation, and research findings from intervention studies targeting frail older people [3]. A geriatric assessment is performed at the emergency ward of the hospital, by registered nurses with geriatric competence. A case manager (CM, a registered nurse) at the municipality is informed of the assessment, in order to start planning the home care. The CM, together with an inter-professional team (consisting of a social worker, an occupational therapist and/or a physiotherapist), performs the planning of the health/social care programme and rehabilitation in the older person's home after that person's discharge from hospital. The CM and the team then continuously follow-up the situation of the older person. The general aim of the model is to create a tight and connected ‘chain of care’ across organisational boundaries, and to deliver integrated health/social care and rehabilitation in the municipality in order to eliminate the need to seek emergency care at the hospital.

## Understanding

### Designing in dialogue

During the interviews it became obvious that the way of working where researchers and practitioners jointly design a new model was familiar to representatives from the municipality but it was unknown and even confusing to the interviewees from the hospital and primary health care.

For a long time I was very frustrated. ‘Why don’t they just tell us what to do?' I want to know how it's supposed to be and then plan according to that. But it never happened that way. (Upper manager, phase 1)

At the upper management level, the representatives from the hospital and primary health care felt that a research project ought to be designed by the researchers. By contrast, the municipal upper managers, who were used to designing development projects in their organisation, were positive about being involved in the design of this project.

In the initial phase of the project, the upper managers and researchers in cooperation supported the operative performance by straightening out problems and questions from the operative staff. Gradually, more and more questions were solved at the operative level and finally the staff established a work system adapted to the local context.

### Reaching out

In the initial phase of the project, the interviewees reported that both the managers and the staff from the units directly involved had been informed about the new model. Furthermore, managers in the municipality responsible for units indirectly affected by the project had been informed. At the hospital the upper managers directly involved in the project and the staff from the emergency ward had been informed. However, staff in the organisational units involved in a more indirect way seemed to have been forgotten in the information process, both in the municipality and at the hospital. Examples were the home care workers at the municipality and the staff from the geriatric and medical wards at the hospital.

They [home help services] came in very late in the process, so we have to put in an effort to reach out to them and get them on board properly. We think they're very important. (Operative staff, phase 1)

As soon as they became aware of this situation, the municipal team and the geriatric nurses at the emergency ward of the hospital acted in order to spread the information to all concerned parties. In the interviews in the middle phases of the project (phases 2 and 3), the information seems to have reached out further within the organisations. Yet in the final phase of the project, the operative staff of the project stressed the importance of continuously providing information about the project to keep it ‘alive’.

### Putting the general aims into practice

Knowledge about the general aims of the introduced model and their association with the performance of practical work emerged as very important in the initial interviews with staff at the operative level. The staff in the municipal team said they believed that the goals would best be achieved by offering accessible and wide-ranging support according to the older people's needs, and by being available for them and their next of kin if they had any questions or worries about the care.

The geriatric nurses in the hospital's emergency ward found it more difficult to link their work according to the model with the general aims. However, in the final phase of the project they talked about the positive feedback they had received from the municipal team about the importance of their geriatric assessment for the work carried out in the municipality. This made the connection to the aim of the model as a whole more obvious to them.

## Commitment

### Links to earlier or parallel projects

In the interviews with the upper managers it became obvious that their commitment to the introduced model was strongly related to earlier projects. Within the hospital, a project with geriatric nurses at the emergency ward had ended some years previously for financial reasons, and the interviewees reported positive experiences. At the municipality, a project involving inter-professional teams performing needs assessments in discharge planning conferences at the hospital had been transformed into a regular model. The interviewees now wanted to contribute their experiences to the new model.

We have experience from 4 years ago, when we had geriatric nurses // at the emergency ward //. And we had very good experiences from that but the financial issue put an end to that. (Upper manager, phase 1)

In a parallel project in primary health care, general practitioners' (GPs') home visits to older patients in need of acute assessment were introduced. This project was shown to be important in the later phases. When the municipal team began to use this as part of the model, primary health care was indirectly involved.

In the interviews with the managers in the municipality during the middle phases it became evident that their loyalty was first and foremost to models introduced before this project concerning the same target group or aiming to solve related issues. The new model was therefore regarded as a rival. At the final phase, new (competing) projects responding to new policies (user choice) were introduced by the upper management.

### What's in it for us?

The interviewees' commitment was closely connected to the potential benefit of the model for their organisation. The representatives from the hospital stressed that the main problem was that many elders repeatedly seek care at the emergency ward. They expressed the hope that a different way of meeting the older people's needs in the municipality could reduce these visits. The benefits of identifying frail older people as a distinct group of patients within the hospital were also mentioned in the interviews.

Regrettably, it is still the last solution of all sorts of things, to be admitted to hospital. (Upper manager, phase 1)

The new model imposes a substantial responsibility on the municipality; however, they expressed the hope that it would provide a good start to the contact between the older people and the municipal team. Performing the needs assessments in the home of the elders ensured a better outcome. The focus on reducing older people's visits to the emergency ward was not very appealing to all. Some interviewees said they would rather see an opportunity for the GPs in primary health care to refer the older people directly to the hospital's medical or geriatric wards.

If a GP in primary health care has sent the patient to hospital and all agree that she or he should be admitted, and it's during daytime, it's difficult to see what they have to do at the emergency ward at all. (Upper manager, phase 1)

Among the upper managers the commitment to the model primarily had to do with economic effectiveness. However, at the operative level the commitment was closely linked to the ideology of care. The operative staff related experiences of when the needs of older people had been neglected both at the hospital and in the municipality, and welcomed the possibility to work in an integrated instead of fragmented manner.

I have been at many places in health care and I've seen that ‘the chain of care’ doesn’t work at all the way you want it to. (Operative staff, phase 1)

In the middle phases, both the upper managers and the operative team in the municipality gave examples of specific cases where the new model had had a significant impact. Several interviewees mentioned that the teamwork allowed them to take a holistic approach to the needs of the older people. The team from the municipality emphasised the importance of facilitating the older people’s contact with primary health care. They said that many elders have difficulties getting in contact with their GP, and the CM in the team used her influence to assist them in this.

The collaboration between the municipal team and the geriatric nurses at the emergency ward was mentioned as being successful and important. The information from the geriatric assessment was stressed as very valuable, especially since it contained more than the standard medical information.

We can follow them [the older people] from the emergency department and are updated all the way. (Operative staff, phase 2)

The representatives from the hospital were more cautious to draw any conclusions regarding the model. However, the geriatric nurses at the emergency ward emphasised the importance of the municipal CM for the hospital staff. To have someone to turn to at the municipality, who is in charge and informed about ‘the whole picture’, made them feel confident that the older people would be taken care of after discharge.

During the final phase of the project, the managers at all levels at the municipality highlighted the fact that quite a few of the older people included in the project needed home nursing or extensive home care from the municipality. Therefore, the model was not being tested on the intended target group, and the municipality offered the services of the CM and the team to older people who were actually the responsibility of primary health care. They stressed that this ‘gap’ needed to be taken care of if the model was to be implemented at full scale.

The most difficultly ill, the most frail that we really wanted to reach, are not included in the project. (Manager, phase 3)

According to the operative team, on the other hand, experiences from the final phase indicated that the intended target group was being reached. They also said that many of the included elders now had increased need of care.

## Ability

### Operative collaboration

In the initial phase, all upper managers emphasised the importance of a smooth collaboration within the municipal team and among different units within the organisations, as well as between the different organisations involved. To succeed in this, all operative staff involved need to have confidence in each other, and also be flexible and creative.

Conditions both promoting and prohibiting smooth cooperation were identified in the middle phases. All operative staff stressed the importance of project meetings at which they all participated regardless of organisational affiliation. These meetings constituted a forum for exchanging experiences and knowledge across professional and organisational boundaries, and promote their collaboration. The operative staff at the municipality emphasised the importance of ‘sitting under the same roof’ to facilitate the team’s collaboration.

The operative collaboration was, according to interviewees, negatively affected by unequal terms for the collaboration. At the municipality, not all members of the team had the same opportunity to read and write in the elders’ social service and municipal health care records. In addition, initially the rehabilitation staff in the team had less time than the other professions, which prevented their full participation in the team. Unequal terms in the cooperation between the hospital and the municipality were mentioned as well. The hospital staff expressed concern that some older patients were being discharged when no-one at the municipality was available to receive information:

Here is a difficulty, we [the hospital] work 24 hours a day but all municipal services close at 4.30 pm. (Manager, phase 4)

In the middle phases of the project, many interviewees stressed the importance of active involvement from primary health care to create a continuum of care for older people. Some representatives from the municipality mentioned the possibility of having a CM in authority to work across both municipal care and services and primary health care.

One may at least work with primary health care and the municipality, of that I’m pretty sure. (Upper manager, phase 4)

### The dilemma of uncertainty

A key issue raised in the interviews was how detailed the guidelines of the model should be. At the operative level, the members of the municipal team were fairly pleased with the freedom to evolve the model on the basis of the general aims of the project, the specific organisational circumstances and the needs of the older people and their relatives. They said it was difficult to think of and work out everything before hand.

It’s all about being flexible and finding solutions together. (Operative staff, phase 2)

The hospital staff, on the other hand, initially had problems with having no specific instructions about the work they were expected to perform. They spent a great deal of time finding out what they were supposed to do and finding the appropriate instrument for their geriatric assessment. In the middle phases of the project, they revised the assessment instrument because they found it too wide-ranging and time-consuming for day-to-day work in practice. What was initially experienced as negative finally came to be valued, however, as interviewees became aware of the benefits of the freedom to create a way of working according to the practical circumstances at hand.

### Gaining acceptance within the organisation

Initially, it was mainly the representatives from the municipality at all levels who stressed the importance of gaining acceptance for the model in the organisation. They all emphasised that the model had the potential to improve care for frail older people. However, they reported that some of their colleagues were very hesitant to adopt the new model since it involved changes to well-established professional roles and working methods.

There has been some resistance among [the ordinary] discharge planning team, since they did this so well and they do it so well. But this is the next step. (Upper manager, phase 3)

The interviewees reported open as well as avoided conflicts. The model gives the CM extended authority normally ascribed to two different categories of home nurses, which caused open conflict. The social workers and their manager were sceptical about the model since some of the social workers’ normal responsibilities now devolved upon the CM. The rehabilitation staff in the team initially did not assess the rehabilitation needs of the elders as intended, and thus avoided conflict. By and by, this changed and about 1 year after introduction of the project they worked according to the model, and even performed some rehabilitation work. In the middle phases, they said that they believed the model had been accepted within the organisation, as a project that ran alongside the ordinary ways of working.

During the interviews in the middle phases of the project, some scepticism among the management in the municipality became evident. The model had been worked out by the upper management in the organisation, but the managers directly responsible for the ‘ordinary’ nurses, social workers and rehabilitation staffs were not convinced of its benefits. These managers are key actors for the model to be fully implemented, and they stated that an unequivocal decision needed to be taken at the top level of the organisation if they should promote the model.

This has to be a decision at the top level of the authority. I think. Maybe even the politicians have to participate and lead the way here: “This is how we are supposed to work.” (Manager, phase 3)

During the interviews in the last phase of the project, most notably at the upper management level both at the hospital and at the municipality, the model’s future and strengths were discussed. All interviewees realised that the model had to be proven to be economically viable for it to survive.

During the interviews at the hospital it became clear that the low status of older patients and the lack of geriatric competence prevented the model from having the desired impact at the emergency ward. The geriatric nurses stressed the importance of having support from the hospital managers to have an impact on the care routines for older patients. Furthermore, the hospital physicians expressed doubt about discharging patients without ‘proper’ discharge planning at the hospital, while the ward nurses said they disliked the many phone calls with the CM and municipal team, which were necessary before discharge.

### Required resources

In the interviews at the operative level at the hospital, the importance of basic material resources was emphasised in the initial phase. The importance of accessing a room, a telephone and a computer with patient care records is understood but these basic requirements had not yet been arranged when the geriatric nurses were appointed to their job. The upper managers of the hospital emphasised the importance of geriatric competence of the geriatric nurses at the emergency ward.

At the municipality, upper managers recruit team members on the basis of previous experience of project and collaborative work. The CM has a key role in the model, and in the interviews at all levels in the municipality the necessary professional background of the CM was discussed. Two viewpoints were revealed, one emphasising the importance of a registered nurse in this position being able to gain acceptance in the health care organisations. The other viewpoint emphasised that the key task of the CM is to coordinate the care, and that any profession in the municipal team can be appointed as CM.

The operative staff initially stressed the importance of enough time to be available for the older people and their relatives. In the middle phases of the project, the need for time assigned to internal teamwork was underlined. Also, interviewees requested a scheduling system for the home care services that fits with the model:

We were supposed to plan the services with home care at an earlier stage but it hasn’t turned out that way because it is their scheduling system that determines that, how they make their schedules. // They can’t plan anything before they know the exact day. (Operative staff, phase 2.)

At the hospital it was observed that the model does not follow the formal guidelines of joint care planning between the hospital and the municipalities, an issue that would need to be addressed in a future, full-scale implementation of the model. Furthermore, the geriatric nurses stressed the importance of a less time-consuming and more realistic instrument for their geriatric assessment.

It was four pages of multiple-choice questions, but now we raise the problems in a different way, conclude // from five key questions. (Operative staff, phase 4)

## Discussion and conclusion

The result of this study points to some circumstances that are important to take into account when implementing a new policy or model in health and social care. However, this study has limitations. The limited involvement of primary health care in the model is reflected in our research. The primary health care perspective is only reflected in the initial phase. Furthermore, the process of implementing a new model in a complex organisational setting is too wide-ranging to allow this study to capture all relevant aspects.

In the interviews, it became evident that the organisations involved had different traditions and a different understanding of project work. Consequently, the roles of the actors involved in designing and implementing the model have to be clearly defined, including who the senders and receivers are, as well as whether the process is to work top down or bottom up, or both [23].

Understanding the general aims of the model, and the ability to connect these to the operative performance, emerged as important in the interviews with the operative staff involved. Grasping the general idea contributed to the understanding of the weight of one’s own work and positively affected the response to the model. However, not only the staff who are directly involved needed to be informed about the general aims of the model. In order for the model to have an impact, the staff in organisational units that are indirectly involved are just as important. In addition to earlier identified components of successful implementation [24], it is important to elaborate a strategy for spreading and maintaining the information about both the aims of the model and how to work according to the model within the organisations.

When introducing a new model, the senders have to be aware of recent and parallel projects in the organisations, and how these fit in with the new one. Fundamental for the commitment of the actors involved in our study was that the new model corresponded to experienced problems and was perceived as having the capacity to respond to them*.* Furthermore, all organisations and professions involved have to see a potential benefit of the model for their own organisation and/or profession. These findings are also supported by results from previous research [34, 35].

Different aspects of what operative staffs need to be able to put the model into practice emerged in the interviews. It emerged as important for the senders of a new model to have sufficient knowledge about the organisations and professional roles involved to secure adequate competence of the operative staff and anticipate possible problems. Furthermore, they have to obtain resources to make implementation of the model possible. This encompasses both basic material resources, and time for the operative staff to perform their work properly, as well as time and other resources to work out and overcome obstacles in the organisations. Consequently, realistic interventions that fit within the existing conditions have been showed to be more successful [34].

The importance of involving all organisations concerned with the issue at hand have been pointed out in the literature [34, 35]. In our study the interviewees reported of occurring problems related to the non-involvement of primary care. Furthermore, an inter-organisational infra-structure has to be evolved at different organisational levels to promote trust and confidence among the actors [22]. In the project, this was done through the steering group and the project group. Both these groups had participants from all organisations involved.^1^

In our study the model was revised and transformed several times during the course of the project. This points to the importance of enabling the operative staff to elaborate the detailed components during the implementation process and adapt the model to the specific circumstances at hand, i.e., to use their discretion [26] guided by mutual aims. Complex interventions have been found to be difficult to implement without allowing some flexibility, but the quality of the implementation relies on the understanding of the underlying ideas and core components of the implemented model [24, 36]. However, staffs operate in a professional landscape of conflicting and competing interests regarding professional status, discretion and jurisdiction [37]. As shown in other studies, our results reveal some conflicts and problems in the implementation process based on professional self-interest [8].

The commitment of the participants is crucial to the impact of the model, and depends on the model’s potential to solve critical problems experienced by the actors involved, professional groups and organisational units as well as whole organisations [34]. What the problems are, and how they are apprehended, is closely related to the demands of the organisation’s environment, and differs according to the organisational level [25, 38]. For the operative staff, the demands came mainly from the older people, their next of kin and the personnel carrying out their health/social care and rehabilitation, and concerned the ideology of care. For the managers, the demands came from both the top levels in their organisation and from the organisation’s environment, and concerned cost and work effectiveness to promote the legitimacy of the organisation [9, 25, 38]. Towards the end of this project, however, new competing ideas and policies regarding user choice caught the municipal managers’ attention and the project was marginalised within the organisation.

The new model comprised changing the traditional working modes and professional and organisational roles, as well as power and status conditions. Organisations, which have been described as a continuously changing and negotiated order [28], consist of segmented social units with contradictory interests and goals. Consequently, the new model imposed a threat to the negotiated order and was partially counteracted and fought by professional as well as organisational actors at different levels [8, 22].

As pointed out by others, the role of upper management in the organisations involved emerged as crucial to the possibility of introducing organisational change, according to the model [34, 35]. Their support for the model had both a symbolic and a factual meaning, and was therefore necessary, although it was affected by factors in the organisation’s environment, such as new, competing ideas and trends. However, to introduce a model top down is a hazardous project. Therefore, a new model that is being introduced has to be experienced as effectively dealing with real problems in the everyday practice of all the actors involved in the organisation, from the bottom to the top. Conflicting conceptions of the problems as well as prioritised goals may hinder the possibility of reaching consensus on the importance of introducing a certain model. Therefore, it is necessary to work out ways to support change among those involved [8].

## Figures and Tables

**Table 1.**
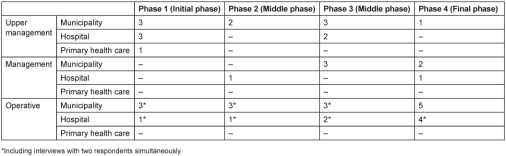
Number of interviews in the different project phases, according to organisational affiliation and level

**Table 2.**
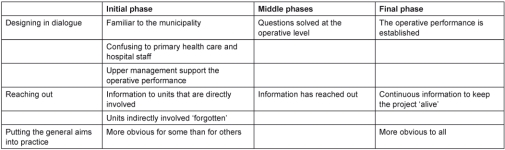
Understanding: themes and subthemes organised according to the project phases

**Table 3.**
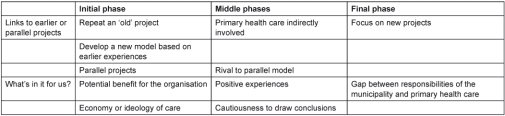
Commitment: themes and subthemes organised according to the project phases

**Table 4.**
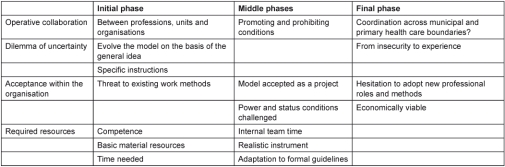
Ability: themes and subthemes organised according to the project phases

## Footnotes

1 In the project group there was no representative from primary health care, since they did not have a formal, active operative part in the project.

## Reviewers

**Carina Abrahamson Löfström**, PhD, Researcher, The Göteborg Region Association of Local Authorities, Sweden

**Bibi Hølge-Hazelton**, Director of Research Region Zealand DK; Honorary Teaching Fellow, Faculty of Health and Life Sciences, Coventry University UK; Associate Research Professor. The Research Unit for General Practice and Section of General Practice, Department of Public Health, University of Copenhagen, Øster Farimagsgade 5 Postboks 2099, 1014 København, Denmark

**Jens Søndergaard**, MD, PhD, Professor, head of research unit, General practitioner, clinical pharmacologist, University of Southern Denmark, Research Unit for General Practice, J.B. Winsløws Vej 9A, 5000 Odense C, Denmark
